# Rapid and Successful Treatment of Interstitial Granulomatous Dermatitis With Tofacitinib: A Case Report

**DOI:** 10.1002/ccr3.73159

**Published:** 2026-07-13

**Authors:** Aida Farmani, Mina Saber

**Affiliations:** ^1^ Department of Dermatology, Skin Diseases and Leishmaniasis Research Center School of Medicine, Isfahan University of Medical Sciences Isfahan Iran

**Keywords:** interstitial granulomatous dermatitis, Janus kinase inhibitors, tofacitinib, tyrosine kinase inhibitors

## Abstract

Interstitial Granulomatous Dermatitis (IGD) is a rare skin condition characterized by granulomatous inflammation. Despite various treatment options, the rarity of this condition makes management challenging. This report highlights the potential of Janus kinase inhibitors, specifically Tofacitinib, as an effective therapy for IGD.

## Key Clinical Message

Interstitial Granulomatous Dermatitis (IGD) is a rare granulomatous inflammatory cutaneous disorder with challenging therapeutic options. This study highlights the potential role of Janus kinase inhibitors, specifically Tofacitinib, as an effective treatment for IGD.

## 1 Introduction

Interstitial Granulomatous Dermatitis (IGD) is a rare skin condition characterized by granulomatous inflammation [1]. Its clinical manifestations include several patterns of skin rash, mostly consisting of asymptomatic patches, papules, and plaques of varying colors and sizes, which can distribute symmetrically on the trunk and proximal limbs [2]. Histopathology reveals focal collagen degeneration, as well as perivascular and interstitial infiltration by lymphocytes and histiocytes [3]. Several conditions, including autoimmune disease, are mentioned in association with IGD [2]. Despite various treatment options, the rarity of this condition makes management challenging. Previous studies have emphasized the importance of controlling the underlying disorder. Hydroxychloroquine, Dapsone, corticosteroids, disease‐modifying antirheumatic drugs, and biological agents are mentioned as effective treatments [4, 5, 6]. According to recent studies, Janus kinase inhibitors work by targeting the JAK–STAT signaling pathway, thereby reducing inflammation in granulomatous diseases such as granuloma annulare, sarcoidosis, and reactive granulomatous dermatitis [7, 8].

## 2 Case History and Examination

**FIGURE 1:**
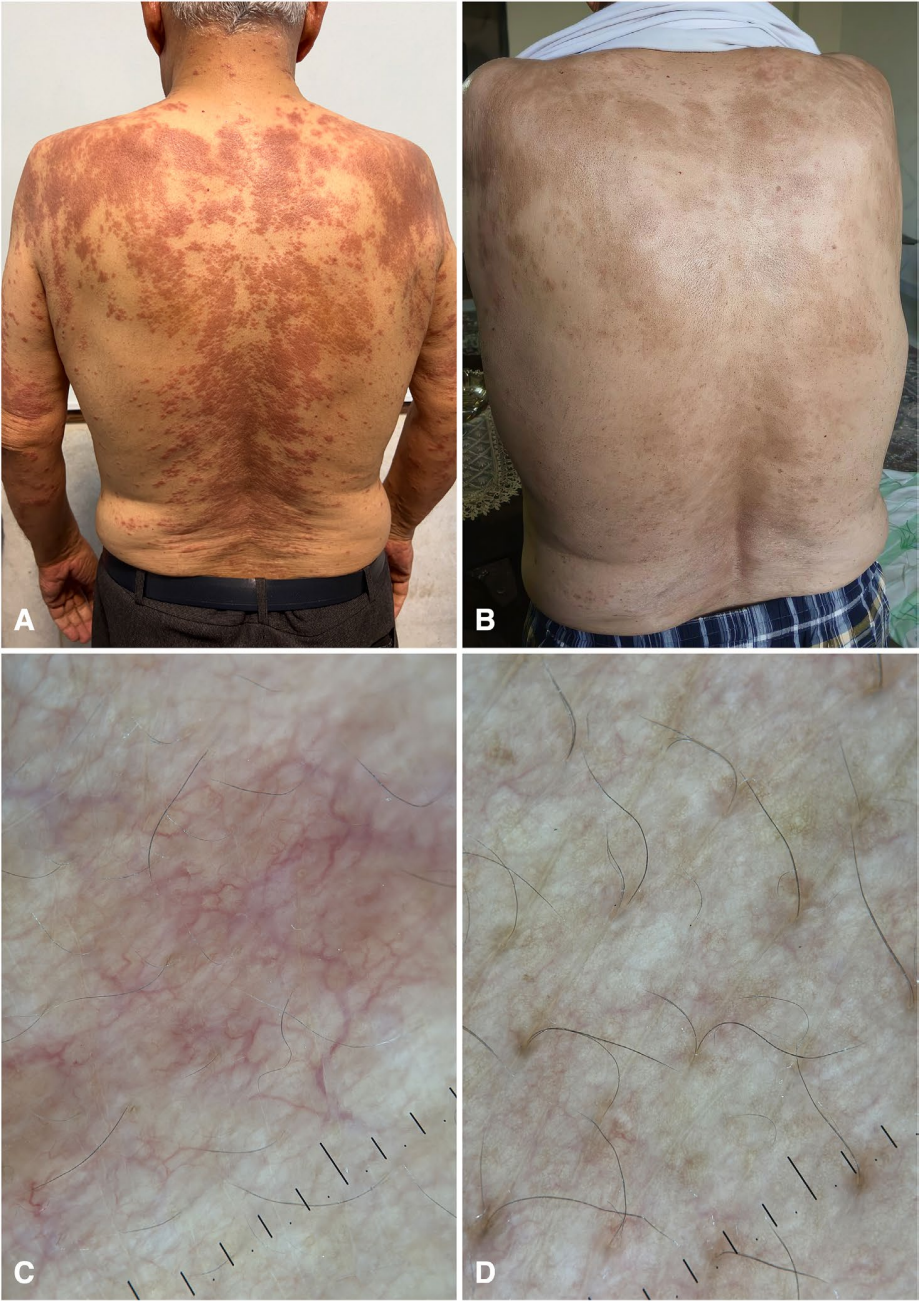
Clinical and dermoscopic pictures of interstitial granulomatous dermatitis. A 63‐year‐old man with a history of 3 months of symmetric, slightly pruritic, and erythematous papular eruption on his back and proximal extremities (A); 3 weeks later, after 21 days of treatment with Tofacitinib (B). Dermoscopy before treatment reveals linear, irregular, approximately sharp vessels on the yellow‐pinkish background (C). After treatment, fading of vascular structures and the pigment network is present, with follicular accentuation (D).

**FIGURE 2:**
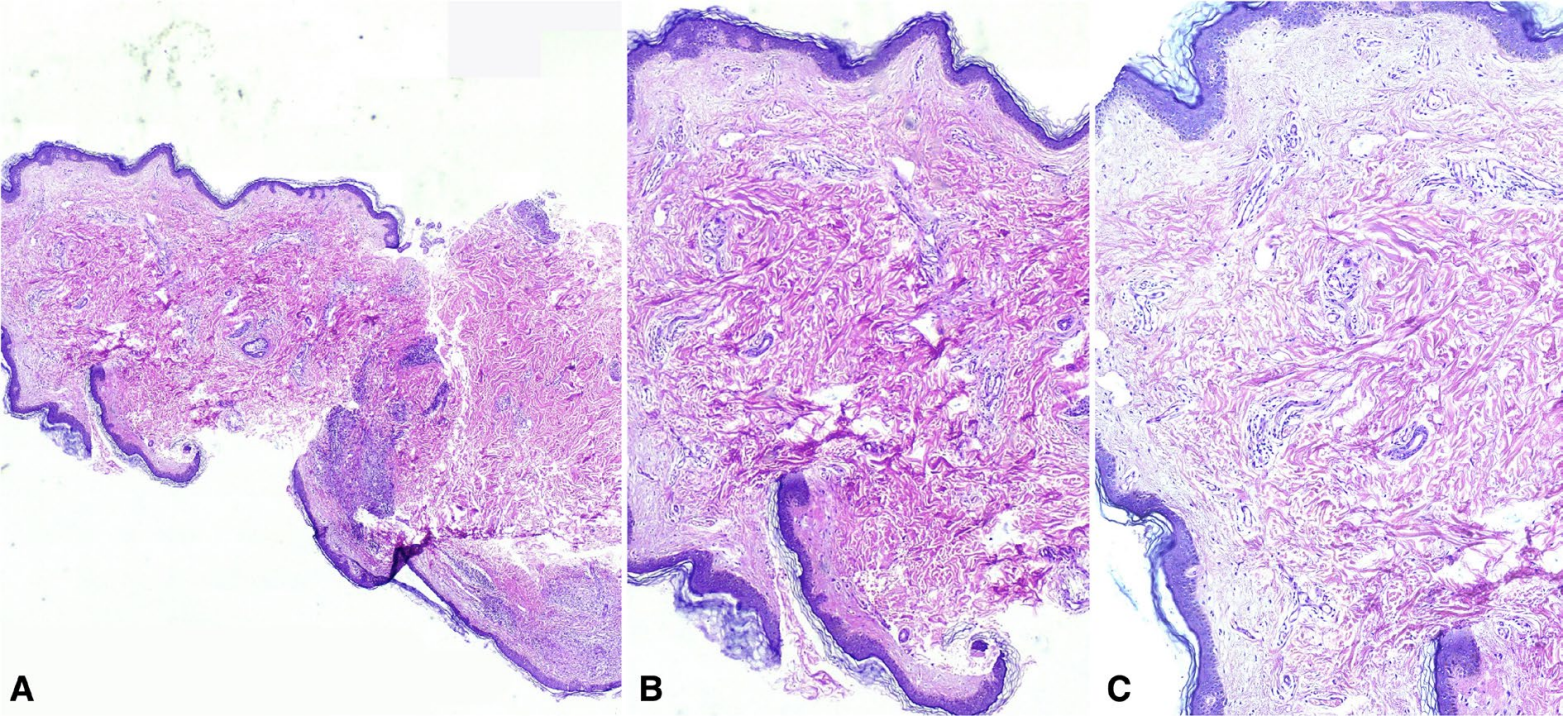
Pathological images reveal superficial and deep perivascular and interstitial infiltrates composed of histiocytes, lymphocytes, rare eosinophils, and giant cells (A, B). Higher magnification displays multifocal areas of degeneration of collagen bundles (C).

A 63‐year‐old male patient was referred to the dermatology outpatient clinic with a history of a 3‐month symmetric, slightly pruritic papular eruption on his back and proximal extremities, without mentioning any therapy. He had a medical history of Chronic Obstructive Pulmonary Disease; however, no treatment was administered. Cutaneous examination revealed multiple discrete erythematous plaques and papules (Figure 1A). The patient was evaluated for possible underlying conditions, including autoimmune disease such as systemic lupus erythematosus, rheumatoid arthritis, and autoimmune thyroiditis; however, none were identified. Laboratory investigations revealed FBS within the prediabetic range, and FANA was positive (speckled pattern). Dermoscopy displays focused linear, irregular, thin, and thick vessels on the yellow‐pink background (Figure 1C). The biopsy reveals dermis with multifocal degeneration of collagen bundles and a superficial and deep perivascular and interstitial infiltration composed of histiocytes, lymphocytes, rare eosinophils, and giant cells. The inflammatory infiltrates extend to some areas of subcutaneous tissue. These histopathological findings confirm the diagnosis of IGD. (Figure 2A–C).

## 3 Differential Diagnosis, Investigations, and Treatment

The main differential diagnoses were granuloma annulare and sarcoidosis [9, 10]. Clinical, dermoscopic, and pathological evaluations ruled them out. The patient was initially treated with hydroxychloroquine 400 mg twice daily for 3 months without significant clinical improvement. Due to a lack of response, the medication was discontinued, followed by a two‐month treatment‐free interval. Then the patient was started on Tofacitinib at a dose of 5 mg twice daily. After 3 weeks of treatment, the follow‐up examination showed only post‐inflammatory hyperpigmentation without primary papules or plaques (Figure 1B). Dermoscopy also revealed network pigmentation, fading of vascular structures, and follicular accentuation (Figure 1D).

Four weeks after treatment initiation, mild thrombocytopenia developed, necessitating dose reduction and eventual discontinuation of tofacitinib. Therapy was not reinitiated. At the 6‐month follow‐up, the patient reported only intermittent papular lesions, which responded well to topical corticosteroids. No further adverse effects or treatment‐related complaints were noted during follow‐up.

## 4 Conclusion and Results

This report highlights the potential role of Janus kinase inhibitors, specifically Tofacitinib, as an effective therapy for IGD.

## 5 Discussion

Interstitial Granulomatous Dermatitis (IGD) is a rare cutaneous condition. Clinical manifestations include asymptomatic patches, papules, and plaques, which are mostly distributed symmetrically on the trunk and proximal limbs [2]. Histopathology reveals infiltration of macrophages surrounding degenerated collagen fibers; diagnosis requires clinicopathologic correlation [1, 10]. This condition has been observed in association with autoimmune disorders, medication use such as TNF‐a inhibitors, malignancies, particularly hematologic neoplasms, infectious disease, and in some cases as an incidental finding [6]. Management of this disease primarily involves controlling the underlying condition, which plays a key role in both improvement and prevention of recurrence [6]. The therapeutic approaches reported for this disease include topical and systemic steroids, dapsone, disease‐modifying antirheumatic drugs, and biologic agents, all of which have demonstrated variable levels of improvement [6]. The pathogenesis of Interstitial Granulomatous Dermatitis is largely unexplored. However, evidence from other granulomatous diseases, namely sarcoidosis, granuloma annulare, and necrobiosis lipoidica, suggests that it has been proposed that inflammatory cytokines, including interleukin‐2 and interferon‐gamma, recruit macrophages to the affected site, which subsequently leads to the phosphorylation of STAT1 and STAT3 [11]. Damsky et al. report that patients with sarcoidosis who were treated with tofacitinib 5 mg twice daily showed a reduction in phosphorylated STAT levels in the lesions and significant improvement of skin and systemic lesions [12, 13, 14, 15] In other studies, a case of generalized granuloma annulare was successfully treated with tofacitinib at a dose of 5 mg twice daily. Additionally, a case report described the use of 2% tofacitinib ointment with noticeable improvement after 15 weeks of treatment [11, 16].

Although the exact pathogenesis of interstitial granulomatous dermatitis (IGD) remains unclear, it is thought to involve immune dysregulation with a key role for pro‐inflammatory cytokines. Many of these cytokines signal through the JAK–STAT pathway, which is essential in regulating inflammatory and immune responses. Inhibition of this pathway by agents such as tofacitinib may therefore reduce cytokine‐mediated immune activation and inflammatory cell infiltration, leading to clinical improvement. This mechanism has been supported by evidence from inflammatory dermatoses that respond to JAK inhibitors, as well as experimental data on cytokine signaling pathways [7, 8, 12, 13, 14, 15]. Reports describing the use of tofacitinib in interstitial granulomatous dermatitis remain limited. Our case is unique for its rapid response within 4 weeks of treatment initiation. Despite the disease's treatment‐resistant nature and extensive skin involvement, with numerous disseminated papules at multiple body sites, the patient showed marked clinical improvement.

Previous reports of granulomatous dermatoses treated with JAK inhibitors have described substantial clinical improvement occurring over subsequent weeks to months. The rapid response observed in our patient further supports the potential role of JAK inhibition in the management of IGD and suggests that tofacitinib may be an effective treatment option for extensive and refractory disease.

Further investigations are required to clarify the meticulous role of JAK inhibitors in the management of granulomatous dermatosis.

## Author Contributions

**Aida Farmani:** conceptualization, data curation, project administration, writing – original draft, writing – review and editing. **Mina Saber:** data curation, methodology, supervision, writing – review and editing.

## Funding

The authors have nothing to report.

## Ethics Statement

This study was conducted in accordance with the principles outlined in the Declaration of Helsinki of the World Medical Association. It was approved by the Ethics Committee of the Isfahan University of Medical Sciences (approval code: IR.ARI.MUI.REC.1404.258).

## Consent

Written informed consent was obtained from the patient for the publication of this case report and accompanying images.

## Conflicts of Interest

The authors declare no conflicts of interest.

## Data Availability

All data supporting the findings of this case report are included in this article. Further details are available from the corresponding author upon reasonable request.
